# Spectrum of Somatic Cancer Gene Variations Among Adults With Appendiceal Cancer by Age at Disease Onset

**DOI:** 10.1001/jamanetworkopen.2020.28644

**Published:** 2020-12-09

**Authors:** Andreana N. Holowatyj, Cathy Eng, Wanqing Wen, Kamran Idrees, Xingyi Guo

**Affiliations:** 1 Department of Medicine, Vanderbilt University Medical Center, Nashville, Tennessee; 2 Vanderbilt-Ingram Cancer Center, Nashville, Tennessee; 3 Department of Surgery, Vanderbilt University Medical Center, Nashville, Tennessee

## Abstract

**Importance:**

The incidence of appendiceal cancer (AC) is rising, particularly among individuals younger than 50 years (early-onset AC), with unexplained etiologies. The unique spectrum of somatic cancer gene variations among patients with early-onset AC is largely undetermined.

**Objective:**

To characterize the frequency of somatic variations and genomic patterns among patients with early-onset (age <50 years) vs late-onset (age ≥50 years) AC.

**Design, Setting, and Participants:**

This cohort study included individuals aged 18 years and older diagnosed with pathologically verified AC. Cases with clinical-grade targeted sequencing data from January 1, 2011, to December 31, 2019, were identified from the international clinicogenomic data-sharing consortium American Association for Cancer Research Project Genomics Evidence Neoplasia Information Exchange (GENIE). Data analysis was conducted from May to September 2020.

**Exposures:**

Age at disease onset.

**Main Outcomes and Measures:**

Somatic variation prevalence and spectrum in AC patients was determined. Variation comparisons between early-onset and late-onset AC were evaluated using multivariable logistic regression with adjustment for sex, race/ethnicity, histological subtype, sequencing center, and sample type.

**Results:**

In total 385 individuals (mean [SD] age at diagnosis, 56.0 [12.4] years; 187 [48.6%] men; 306 [79.5%] non-Hispanic White individuals) with AC were included in this study, and 109 patients (28.3%) were diagnosed with early-onset AC. Race/ethnicity differed by age at disease onset; non-Hispanic Black individuals accounted for a larger proportion of early-onset vs late-onset cases (9 of 109 [8.3%] vs 11 of 276 [4.0%]; *P* = 0.04). Compared with patients aged 50 years or older at diagnosis, patients with early-onset AC had significantly higher odds of presenting with nonsilent variations in *PIK3CA*, *SMAD3*, and *TSC2* (*PIK3CA*: odds ratio [OR], 4.58; 95% CI, 1.72-12.21; *P* = .002; *SMAD3*: OR, 7.37; 95% CI, 1.24-43.87; *P* = .03; *TSC2*: OR, 12.43; 95% CI, 1.03-149.59; *P* = .047). In contrast, patients with early-onset AC had a 60% decreased odds of presenting with *GNAS* nonsilent variations compared with patients with late-onset AC (OR, 0.40; 95% CI, 0.21-0.76, *P* = .006). By histological subtype, young patients with mucinous adenocarcinomas of the appendix had 65% decreased odds of variations in *GNAS* compared with late-onset cases in adjusted models (OR, 0.35; 95% CI, 0.15-0.79; *P* = .01). Similarly, patients with early-onset nonmucinous appendiceal adenocarcinomas had 72% decreased odds of presenting with *GNAS* variations vs late-onset cases, although these findings did not reach significance (OR, 0.28; 95% CI, 0.07-1.14; *P* = .08). *GNAS* and *TP53* variations were mutually exclusive in ACs among early-onset and late-onset cases (*P* < .05).

**Conclusions and Relevance:**

In the study, AC diagnosed among younger individuals harbored a distinct genomic landscape compared with AC diagnosed among older individuals. Development of therapeutic modalities that target these unique molecular features may yield clinical implications specifically for younger patients.

## Key Points

**Question:**

What are the differences in somatic cancer gene variations in appendiceal cancer among adults based on age at disease onset?

**Findings:**

In this cohort study of 385 patients diagnosed with appendiceal cancer with targeted clinical-grade sequencing data from the American Association for Cancer Project Genomics Evidence Neoplasia Information Exchange, patients who were diagnosed at age younger than 50 years harbored unique somatic variant patterns in *PIK3CA*, *GNAS*, *SMAD3*, and *TSC2* compared with those diagnosed at age 50 years and older.

**Meaning:**

These findings suggest that appendiceal cancer diagnosed among young individuals harbors a distinct spectrum of somatic variations, which may yield clinical actionability in the development of targeted therapeutic modalities for young patients with appendiceal cancer.

## Teaser

This cohort study characterizes the frequency of somatic variations and genomic patterns among patients who were diagnosed with appendiceal cancer younger than 50 years and those diagnosed at age 50 years or older.

## Introduction

Appendiceal cancer (AC) is a rare neoplasm, with an age-adjusted incidence rate of 0.12 per 1 000 000 person-years.^1,2^ The rarity of AC has presented challenges in understanding disease pathogenesis and in developing clinical management guidelines for AC. Definitive treatment for early-stage AC is surgery, and cytoreductive surgery (CRS) as well as the consideration of heated intraperitoneal chemotherapy (HIPEC) may also yield long-term survival benefit for select patients. However, most patients will present with distant metastatic disease with significant tumor burden in the peritoneum, placing them at higher risk for bowel obstruction and increased morbidity and mortality. For most patients with AC, CRS and HIPEC are not feasible, and systemic chemotherapy will be provided only for palliative intent. Currently, the National Comprehensive Cancer Network guidelines recommend treatment of AC cases with systemic therapy according to colon cancer guidelines.^3^ This is largely because of lack of robust data for AC, and treatment regimens are extrapolated from clinical studies related to colon cancer. However, emerging evidence reveals distinct molecular features between colorectal cancer (CRC) and AC.^4,5,6,7^ Recent genomic profiling of AC has begun to shed light on distinct variant profiles among patients of all ages, given that *GNAS* (OMIM 139320) and *TP53* (OMIM 191170) variations were associated with overall survival.^8^ However, earlier studies reported contradictory findings because *GNAS* variations were not associated with survival among patients with appendiceal mucinous neoplasms.^9^ In the absence of prognostic and predictive biomarkers and new therapeutic targets specific to AC, therapeutic advances in this malignant neoplasm remain very limited.

Given the rarity of AC, little is also known regarding risk factors and the epidemiology of this disease. Incidence rates of individuals of all ages with malignant AC have risen 232% between 2000 and 2016 in the United States.^10,11^ However, rates of appendectomies—where many AC cases are detected as incidental findings^12,13^—remained stable during this period.^11^ Given that AC incidence rates also continue to rise in older and younger patients,^11^ these findings have raised the question of what causes underlie the rising burden of AC among patients diagnosed younger than 50 years (ie, early-onset AC). Our recent findings^14^ have shed light on the clinicopathologic and demographic patterns of early-onset AC, noting disparities in survival among young patients by race/ethnicity and sex. However, to our knowledge, no studies to date have compared molecular phenotypes of AC by age at disease onset. Given the known molecular phenotypes unique to early-onset vs late-onset CRC,^15,16^ we hypothesized that distinct etiologies also underlie the growing AC burden among young patients. The purpose of this study, comprised of patients from the international clinicogenomic data-sharing consortium American Association of Cancer Research (AACR) Project Genomics Evidence Neoplasia Information Exchange (GENIE),^17^ was to characterize distinct putative driver variations and genes between patients diagnosed with early-onset and late-onset AC.

## Methods

### Data Sources and Study Population

The AACR GENIE project^17^ has generated next-generation clinical sequencing data in tumor tissues and associated pathology reports from multiple cancer centers in the United States, Canada, and Europe. This study has been granted data access through Database of Genotypes and Phenotypes (dbGap) project #24541. Somatic variation and clinical data from AC cases were downloaded from the GENIE project via Synapse (release 7).^18^ This study was exempt from institutional review board approval and informed consent because deidentified GENIE data are publicly available to the entire scientific community.^17^ This study followed the Strengthening the Reporting of Observational Studies in Epidemiology (STROBE) reporting guideline. A total of 385 pathologically confirmed AC cases with a unique patient record and matched clinical and variation data sequenced between January 1, 2011, and December 31, 2019, were included in our study.

### Clinicopathologic and Demographic Features

Demographic variables examined included patient sex, age at diagnosis, race/ethnicity (non-Hispanic White, non-Hispanic Black, Hispanic/Spanish/Latino, Asian or Pacific Islander, or other), and sequencing center. Clinical and pathological variables examined included histological subtype (nonmucinous adenocarcinoma, mucinous adenocarcinoma, goblet cell carcinoid, and signet ring cell carcinoma) and sample type (primary tumor or metastatic site).

### Somatic Cancer Gene Variations

Somatic variation data in tumor tissues have been generated using clinical-grade targeted gene panel sequencing approaches from different sequencing centers. Median sequencing depth (pooled median read depth, 500X) by sequencing center is outlined in eTable 1 in the Supplement. To ensure consistent somatic variation calling in tumor tissues and to minimize artifacts and germline events, GENIE has applied a stringent filtering pipeline to remove putative germline variants (eg, using pooled blood samples as controls, existing databases of known artifacts, and common germline variants from the 1000 Genomes Project or Exome Sequencing Project with allele frequencies >0.1%). We restricted our analysis to nonsilent variants, including missense, frameshift, nonframeshift, splicing, nonsense, and truncating variations, defined as frameshift, splicing, and nonsense variations. Nonsilent variation events (eg, bin variable) and variant frequencies were calculated based on study participants harboring at least 1 nonsilent variation, as we have previously described.^19^ A recurrent variation was defined as a nonsilent variant observed in at least 3 patients within our cohort.

### Statistical Analysis

To assess clinical and demographic features between patients diagnosed with early-onset AC (age <50 years) and late-onset AC (age ≥50 years), features were compared by age at disease onset group using χ^2^ or Fisher exact tests for categorical variables and *t* tests for continuous variables. The significance levels of cooccurrence and mutual exclusivity for a pair of variant genes were calculated by the Mutual Exclusivity Modules statistical method from cBioportal.^20^

Variant comparisons by age at AC onset were evaluated using multivariable logistic regression analysis with an adjustment for patient sex, race/ethnicity, histological subtype, sequencing center, and primary sample type, in which all covariates were used as fixed effects and the reference outcome category was individuals diagnosed with late-onset AC. In addition, we performed similar analysis stratified by histological subtype. All tests were 2-sided, and *P* < .05 was considered statistically significant. All analyses were conducted using R software version 3.3.3 (R Project for Statistical Computing).

## Results

A total of 385 individuals diagnosed with AC were identified from 12 international institutions within the AACR Project GENIE Consortium during the 9-year study period (Table 1). Approximately 30% of the population was diagnosed with early-onset AC (109 patients [28.3%]), and mean (SD) age at cancer diagnosis was 56.0 (12.4) years. A total of 187 men (48.6%) were in the sample, and the proportion of men did not differ between early-onset vs late-onset AC cases (54 [49.5%] vs 133 [48.2%]; *P* = .81). Approximately 4 of every 5 patients was a non-Hispanic White individual (306 [79.5%]). Race/ethnicity differed by age of AC onset; non-Hispanic Black patients accounted for a larger proportion of early-onset vs late-onset cases (9 of 109 [8.3%] vs 11 of 276 [4.0%]; *P* = .04). By histological subtype, 177 patients (44.4%) were diagnosed with nonmucinous adenocarcinoma, 156 (40.5%) had mucinous adenocarcinoma, 32 (8.3%) had goblet cell appendiceal carcinoma, and 26 (6.8%) had signet ring cell appendiceal carcinoma (Table 1). However, histological subtype did not statistically significantly differ by age of AC onset in this cohort.

**Table 1.** Clinical and Demographic Characteristics of Patients Diagnosed With Appendiceal Cancer From the American Association of Cancer Research Project Genomics Evidence Neoplasia Information Exchange, 2011 to 2019

Characteristic	No. (%)			*P* value^a^
	Total (N = 385)	Age at appendiceal cancer onset, y		
		<50 (n = 109)	≥50 (n = 276)	
Age at diagnosis, y				
<30	9 (2.3)	9 (8.3)	0	NA
30-39	26 (6.8)	26 (23.9)	0	
40-49	74 (19.2)	74 (67.9)	0	
50-59	125 (32.5)	0	125 (45.3)	
60-69	95 (24.7)	0	95 (34.4)	
70-79	48 (12.5)	0	48 (17.4)	
≥80	8 (2.1)	0	8 (2.9)	
Mean (SD)	56.0 (12.4)	41.2 (7.3)	61.9 (8.4)	NA
Race/ethnicity				
Non-Hispanic				.04
White	306 (79.5)	81 (74.3)	225 (81.5)	
Black	20 (5.2)	9 (8.3)	11 (4.0)	
Hispanic, Spanish, or Latino	13 (3.4)	3 (2.8)	10 (3.6)	
Asian or Pacific Islander	9 (2.3)	6 (5.5)	3 (1.1)	
Other	3 (0.8)	1 (0.9)	2 (0.7)	
Unknown	34 (8.8)	9 (8.3)	25 (9.1)	
Sex				
Women	198 (51.4)	55 (50.5)	143 (51.8)	.81
Men	187 (48.6)	54 (49.5)	133 (48.2)	
Histological subtype				
Adenocarcinoma				.31
Nonmucinous	171 (44.4)	45 (41.3)	126 (45.7)	
Mucinous	156 (40.5)	48 (44.0)	108 (39.1)	
Goblet cell	32 (8.3)	6 (5.5)	26 (9.4)	
Signet ring cell	26 (6.8)	10 (9.2)	16 (5.8)	
Sample type				
Primary tumor	165 (42.9)	45 (41.3)	120 (43.5)	.53
Metastasis	205 (53.2)	62 (56.9)	143 (51.8)	
Unknown	15 (3.9)	2 (1.8)	13 (4.7)	

Abbreviation: NA, not applicable.

^a^ *P* value calculations did not include unknown values.

A total of 39 genes in AC had a variation frequency of greater than 2% among all patients (Figure). More than half of all ACs (198 [51.4%]) had a *KRAS* variation (OMIM 190070), consistent with previous reports (Figure, A).^4,21,22,23^ *TP53* and *GNAS* were altered in more than one-quarter of all ACs (105 [27.3%] and 101 [26.2%], respectively) (Figure, A). Other genes commonly altered in at least 5% of AC cases included *SMAD4 *(OMIM 600993)*, APC *(OMIM 611731)*, PIK3CA *(OMIM 171834),* KMT2D *(OMIM 602113),* SOX9* (OMIM 608160), and *ATM *(OMIM 607585). Patterns of significant gene cooccurrence and mutual exclusivity by age at disease onset are described in Figure, B. Among both early-onset and late-onset AC cases, *GNAS* and *TP53* variations were mutually exclusive (*P* < .05) (Figure, B). Among young patients with AC, *SOX9* and *KRAS* variations as well as *SOX9* and *TP53* variations were also mutually exclusive pairs (*P* < .05). The frequency and type of variations for the top 10 frequently altered genes among ACs in patients diagnosed with early-onset and late-onset disease are presented in Figure, C and D, respectively. In particular, *GNAS* and *PIK3CA* harbored distinct variation frequencies between early-onset and late-onset ACs. A total of 21 of 109 young patients (19.3%) had ACs with *GNAS* variations, whereas nearly one-third of late-onset cases (80 of 276 [29.0%]) has variations in *GNAS* (Figure, C). In contrast, nearly 1 in 8 young patients had ACs with *PIK3CA* variants (13 [11.9%]), while only 13 tumors (4.7%) among patients aged 50 years and older had variants in *PIK3CA* (Figure, D).

**Figure.**
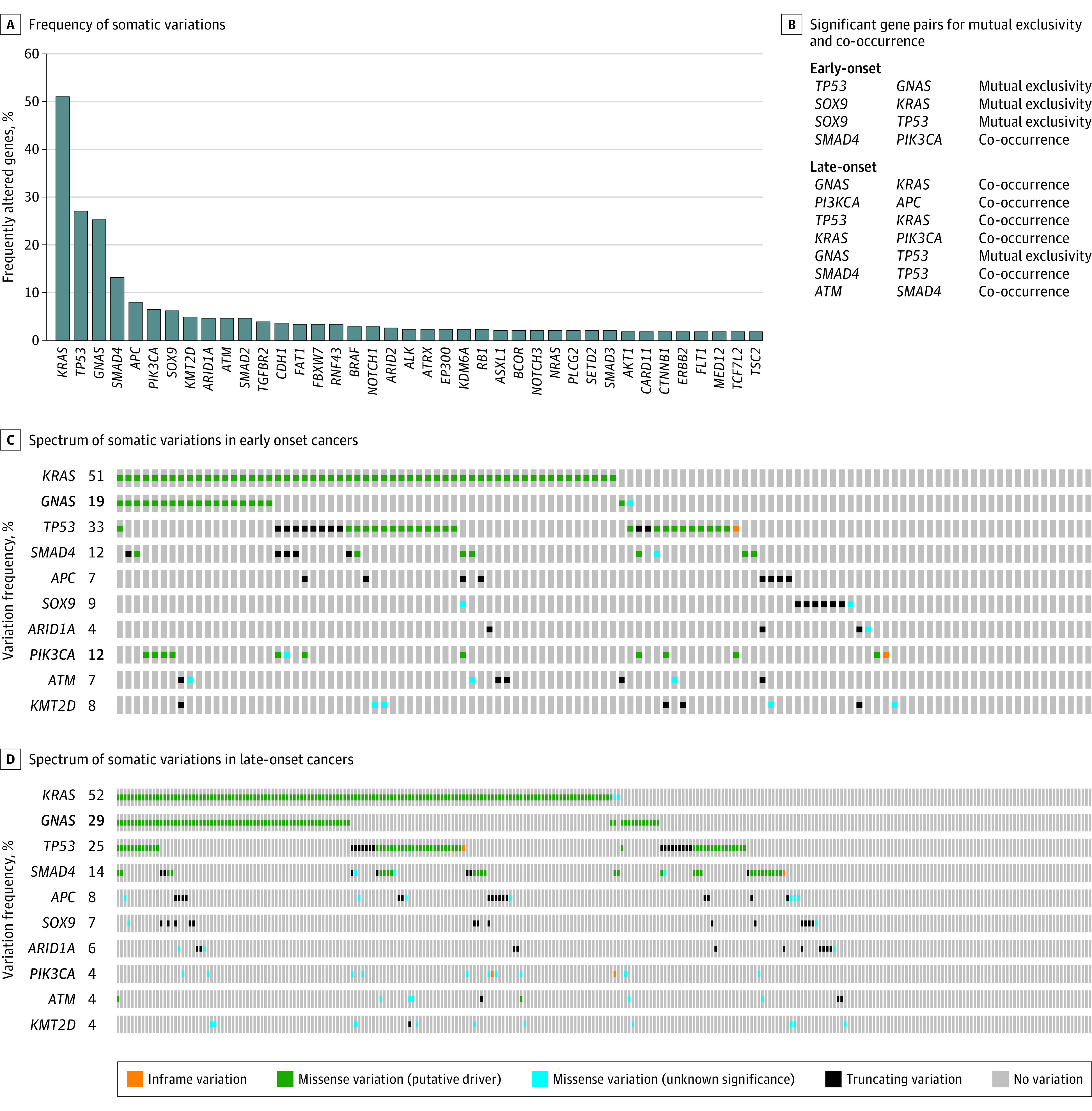
Genomic Landscape of Appendiceal Cancers by Age at Disease Onset

Baseline variation probabilities among all AC patients and by early-onset vs late-onset AC are presented in Table 2. Next, we sought to characterize somatic alterations unique to patients with early-onset vs late-onset ACs. Among all patients with AC, young patients had significantly higher odds of presenting with nonsilent *PIK3CA*, *SMAD3*, and *TSC2* somatic variations in ACs compared with late-onset AC cases after adjustment for sex, race/ethnicity, histological subtype, sequencing center, and sample type (*PIK3CA*: odds ratio [OR], 4.58; 95% CI, 1.72-12.21; *P* = .002; *SMAD3*: OR, 7.37; 95% CI, 1.24-43.87; *P* = .03; *TSC2*: OR, 12.43; 95% CI, 1.03-149.59; *P* = .047) (Table 2). In contrast, young AC patients had 60% decreased odds of presenting with nonsilent *GNAS* variations compared with late-onset cases in adjusted models (OR, 0.40; 95% CI, 0.21-0.76; *P* = .006). Moreover, we observed dominant recurrent nonsilent variations for both *PIK3CA* and *GNAS,* providing additional evidence of their putative role in appendiceal carcinogenesis (Table 2). Notably, our main findings for *PIK3CA* remained statistically significant after adjustment for multiple testing among highly altered (ie, >4%) genes (data not shown).

**Table 2.** Baseline Variation Probability and Differential Expression of Somatic Variants Between Patients With Early-Onset and Late-Onset AC

Gene symbol^a^	Baseline variant probability	Baseline variant probability by age at AC onset, y		OR (95% CI)^b^	*P* value
		<50 y	≥50 y		
*KRAS*	0.5143	0.5229	0.5109	0.98 (0.58-1.66)	.94
*TP53*	0.2734	0.3303	0.2509	1.49 (0.87-2.55)	.15
*GNAS*	0.2630	0.1927	0.2909	0.40 (0.21-0.76)	.006
*SMAD4*	0.1328	0.1193	0.1382	0.95 (0.45-2.04)	.90
*APC*	0.0805	0.0734	0.0833	0.95 (0.38-2.38)	.91
*SOX9*	0.0772	0.0889	0.0724	1.59 (0.61-4.12)	.34
*PIK3CA*	0.0649	0.1193	0.0435	4.58 (1.72-12.21)	.002
*KMT2D*	0.0538	0.0762	0.0444	2.16 (0.71-6.54)	.17
*TGFBR2*	0.0524	0.0330	0.0615	0.54 (0.13-2.20)	.39
*SMAD2*	0.0510	0.0667	0.0444	1.38 (0.47-4.07)	.56
*ARID1A*	0.0510	0.0381	0.0565	0.66 (0.19-2.25)	.50
*ATM*	0.0469	0.0734	0.0364	1.81 (0.61-5.43)	.29
*FAT1*	0.0418	0.0333	0.0452	0.77 (0.19-3.18)	.72
*RNF43*	0.0402	0.0619	0.0310	1.75 (0.50-6.13)	.38
*CDH1*	0.0365	0.0183	0.0436	0.41 (0.07-2.52)	.34
*FBXW7*	0.0339	0.0367	0.0327	1.05 (0.29-3.81)	.94
*NOTCH1*	0.0286	0.0275	0.0291	1.03 (0.19-5.57)	.97
*BRAF*	0.0286	0.0183	0.0326	0.41 (0.06-2.84)	.36
*ARID2*	0.0283	0.0095	0.0363	0.28 (0.03-2.36)	.24
*SMAD3*	0.0257	0.0556	0.0136	7.37 (1.24-43.87)	.03
*EP300*	0.0255	0.0190	0.0282	0.55 (0.08-3.86)	.55
*ATRX*	0.0254	0.0286	0.0240	1.40 (0.26-7.57)	.70
*KDM6A*	0.0254	0.0286	0.0240	1.32 (0.27-6.33)	.73
*NOTCH3*	0.0248	0.0515	0.0133	4.08 (0.88-18.87)	.07
*PLCG2*	0.0248	0.0309	0.0221	1.13 (0.24-5.31)	.88
*BCOR*	0.0246	0.0103	0.0307	0.24 (0.02-2.83)	.26
*RB1*	0.0234	0.0275	0.0218	0.75 (0.13-4.18)	.74
*ALK*	0.0234	0.0092	0.0290	0.21 (0.02-2.14)	.19
*SETD2*	0.0227	0.0476	0.0121	4.03 (0.84-19.41)	.08
*ASXL1*	0.0219	0.0280	0.0194	1.82 (0.32-10.33)	.50
*TCF7L2*	0.0217	0.0323	0.0175	1.90 (0.35-10.48)	.46
*MED12*	0.0216	0.0412	0.0132	3.22 (0.59-17.44)	.17
*NRAS*	0.0208	0.0183	0.0217	1.12 (0.21-6.13)	.89
*TSC2*	0.0198	0.0381	0.0121	12.43 (1.03-149.59)	.047
*CARD11*	0.0198	0.0190	0.0202	0.98 (0.18-5.31)	.98
*FLT1*	0.0198	0.0095	0.0242	1.54 (0.13-18.30)	.73
*ERBB2*	0.0182	0.0367	0.0109	3.27 (0.55-19.45)	.19
*CTNNB1*	0.0182	0.0183	0.0181	1.34 (0.23-7.75)	.74
*AKT1*	0.0182	0.0092	0.0217	0.31 (0.03-3.53)	.34

Abbreviations: AC, appendiceal cancer; OR, odds ratio.

^a^ Genes ranked by baseline probability of variation occurrence.

^b^ ORs and 95% CIs were calculated for genes from models adjusted for patient sex, race/ethnicity, histological subtype, sequencing center, and sample type. Reference outcome category was individuals with late-onset AC.

To further explore age-related somatic cancer gene variation patterns, we evaluated baseline variant probability among individuals aged younger than 50, 50 to 59, 60 to 69, and 70 years or older at AC diagnosis (eTable 2 in the Supplement). Concordant with our findings, baseline variation probabilities of *PIK3CA*, *SMAD3*, and *TSC2* were highest for patients diagnosed with AC younger than 50 years across all age groups. Similarly, baseline *GNAS* variation probability also remained lowest among early-onset AC cases. Additional comparison of somatic cancer gene variation patterns specifically among adults diagnosed with AC younger than 50 years vs those aged 70 years or older revealed consistent findings, given that AC cases among those younger than 50 years had 74% decreased odds of presenting with nonsilent *GNAS* variations (OR, 0.26; 95% CI, 0.11-0.63; *P* = .003) compared with adults aged 70 years or older at AC diagnosis (eTable 2 in the Supplement). Similarly, early-onset AC cases had significantly higher odds of presenting with nonsilent *PIK3CA* variations compared with those aged 70 years or older (OR = 11.69; 95% CI, 1.37-99.82; *P* = .02).

Stratification of patients by histological subtype revealed that young patients with mucinous adenocarcinomas of the appendix had 65% decreased odds of nonsilent variations in *GNAS* (OR, 0.35; 95% CI, 0.15-0.79; *P* = .01) compared with late-onset cases in adjusted models (eTable 3 in the Supplement). Similarly, for patients with non-mucinous appendiceal adenocarcinomas, young individuals had 72% decreased odds of presenting with *GNAS* variations compared with late-onset cases, although these findings were not statistically significant (OR, 0.28; 95% CI, 0.07-1.14; *P* = .08) (eTable 3 in the Supplement).

## Discussion

The genomic landscape of 385 appendiceal neoplasms provides novel insight into molecular differences of AC by age at disease onset and identifies potential biomarkers associated with AC diagnosed at younger ages that may help unravel distinct etiologies underlying the increasing incidence of early-onset AC. Most striking are differences in the variation patterns of *GNAS*, *PIK3CA, TSC2*, and *SMAD3* between early-onset and late-onset AC cases. Compared with cases diagnosed at age 50 years and older, younger patients had higher odds of presenting with somatic variations in *PIK3CA, SMAD3*, and *TSC2*, whereas younger patients had decreased odds of presenting with somatic variations in *GNAS*. Differences in *GNAS* by age at onset were also noted in stratified analyses for cases diagnosed with mucinous adenocarcinomas of the appendix. Moreover, *GNAS* and *TP53* variations were mutually exclusive for ACs among patients with early-onset and late-onset disease.

Pathogenesis of AC is driven by the accumulation of genetic and epigenetic alterations, which remain largely unknown. Somatic variations of *GNAS,* a heterotrimeric G protein α subunit that activates adenylyl cyclase downstream of activated G protein–coupled receptors in response to hormones and a plethora of extracellular signals,^24^ have been identified in many gastrointestinal diseases, including neoplasms of the pancreas^25,26,27,28^ and stomach^29^ as well as adenomas of the colorectum.^30,31^ However, *GNAS* variation patterns in ACs remain incompletely understood. To date, studies have reported conflicting evidence on the prevalence of *GNAS* variants by tumor histological subtype among ACs.^4,9,32^ In a 2018 study of 703 AC samples,^8^ *GNAS* variations were reported in 22% of nonmucinous adenocarcinomas and in 49% of mucinous adenocarcinomas of the appendix. In the present study, we observed that approximately 1 in every 4 appendiceal tumors carried a *GNAS* variation. Among ACs diagnosed in patients with early-onset and late-onset disease, *GNAS* variations were also found to be mutually exclusive with *TP53* variations. Moreover, we reported that younger patients with AC had 63% decreased odds of presenting with *GNAS* variations compared with late-onset cases, patterns that persisted among patients with mucinous adenocarcinomas of the appendix. Given that previous studies have revealed that most high-grade ACs are *GNAS* wild-type tumors and also that *GNAS* and *TP53* variations tend to be mutually exclusive,^7,8^ these findings suggest that a subset of early-onset ACs may be more likely to occur de novo rather than progressing from low-grade tumors—emphasizing that distinct pathways may contribute to early-onset AC. Given this mutual exclusivity for *GNAS* and *TP53* variations and reduced likelihood for young patients with AC to have somatic *GNAS* variations compared with late-onset cases, future studies are also warranted to examine germline *TP53* variants and hereditary syndromes among young patients diagnosed with AC.

*PIK3CA* encodes the p110 catalytic subunit of phosphatidylinositol-3-kinase (PI3K), among the key kinases in PI3K/AKT and the mammalian target of rapamycin (mTOR) (PI3K/AKT/mTOR) signaling,^33,34^ and promotes malignant cell growth and invasion.^35^ *PIK3CA* is among the most commonly altered genes across various cancer types, including CRC and gastric tumors.^36^ A 2019 comparison of *PIK3CA* variation frequencies between appendiceal adenocarcinoma and CRC cases revealed lower variation rates in appendiceal neoplasms (6% vs 17%-22%).^4^ Similar to these and other findings,^4,6,37,38^ *PIK3CA* variations were reported in 6.8% of AC cases in our cohort. However, in contrast to previous results from variation frequencies between patients diagnosed with early-onset vs late-onset CRCs that did not identify differences in *PIK3CA* variation rates,^39^ here we observed distinct *PIK3CA* variation patterns by age at disease onset for AC, which persisted after adjustment for multiple testing among highly altered genes. Compared with late-onset AC cases, young patients had a 4.7-fold increased odds of presenting with *PIK3CA* variations in ACs, findings that persisted after adjustment for multiple testing. These findings provide initial insight to suggest that mechanisms of early-onset appendiceal carcinogenesis may be distinct from early-onset colorectal carcinogenesis. Moreover, as alpelisib—a *PIK3CA* inhibitor—became FDA-approved last year for *PIK3CA*-altered, hormone receptor–positive advanced breast cancer,^40^ this study reveals that 12% of early-onset AC cases could potentially benefit from targeting this variation and merits further study. Moreover, as studies have posited that adolescents and young adults (AYAs; age 18-39 years) harbor a distinct biology of cancer^41,42,43^; additional investigation of variation patterns within the AYA population are needed in larger cohorts.

Currently, the roles of *TSC2* and *SMAD3* in appendiceal carcinogenesis remain unexplored. *TSC2* is a target of RAS/ERK signaling, and direct phosphorylation of tuberous sclerosis complex 2 (TSC2) by ERK leads to suppression of tumor-suppressive functions.^44^ A study of 63 colon carcinomas^45^ showed that approximately one-third of colon carcinomas were positive for phosphorylated TSC2. Moreover, reduced expression of TSC2 was also found to be associated with shorter disease-free survival among 50 patients with CRC.^46^ Notably, TSC2 was shown to positively regulate expression of mucin2, a marker of goblet cell differentiation in intestinal cells.^47,48^ TSC2 inactivation altered differentiation throughout the intestinal epithelium, with a marked decrease in goblet cell lineages.^49^ As goblet cell carcinoid tumors accounted for less than 10% of cases in this cohort, we were unable to assess genomic differences of AC by age at disease onset specific to this histological subtype. Nevertheless, as young patients had higher odds of presenting with *TSC2* variations, these findings posit a potential role for targeting the mTOR network^50^ in AC therapy, particularly for young patients.

*SMAD* genes are key mediators of transforming growth factor β (TGF-β) signals that, on inactivation, enhance tumor growth.^51,52^ Previous studies have reported that *SMAD3* variations are infrequent in CRCs (<5% of sporadic tumors and colorectal liver metastases).^51,53,54,55^ Consistent with these reports, we observed *SMAD3* variations in fewer than 5% of AC cases. Moreover, *SMAD3* variations had higher odds of occurrence in ACs of young patients, positing a potential distinct role for *SMAD3* as well as *TSC2* in early-onset appendiceal carcinogenesis. Given the relatively low somatic variation frequency in *TSC2* and *SMAD3* in our cohort, further investigations are warranted to explore the mechanistic role of these genes and related pathways, particularly in early-onset AC.

### Strengths and Limitations

The use of data from the GENIE clinicogenomic data-sharing consortium is a strength of this study because it allowed for pathologically verified cases with clinical-grade sequencing data to be identified from 12 institutions worldwide. However, we also acknowledge that our study has limitations. Our analyses were conducted using GENIE data from a large number of patients with AC; however, GENIE does not record information about cancer stage, metastasis sites, pseudomyxoma peritonei, or tumor grade (eg, low-grade appendiceal mucinous neoplasms). As such, we were unable to assess for differences in these tumor characteristics by age at AC onset or to investigate whether these differences were associated with distinct genomic patterns of early-onset AC. Similar to previous studies,^8^ specimens submitted for sequencing in GENIE derived from primary ACs and metastatic sites. Given that half of all tumors in this study derived from metastases—with similar proportions for early-onset and late-onset AC cases—these findings are indicative that most patients in this study had stage IV disease. However, primary AC tissue may have been sequenced in cases that presented with metastatic disease, which does not allow us to rule out that the molecular patterns reported in this study may be in part related to AC stage. In addition, because all somatic variations were not systematically evaluated within GENIE, the true prevalence of somatic variations in our cohort may be even higher. Risk of potential bias also exists in our study due to overfitting variations that occur with a small probability.^56,57^ GENIE also lacks detailed information regarding individual-level characteristics, including family history of cancer, and does not provide any data about germline genetic features, cancer treatments, or prognostic outcomes for patients with AC.

## Conclusions

To our knowledge, this international consortium study is the first to examine molecular features of AC by age at disease onset. This study found a distinct spectrum of somatic variations among early-onset AC cases, as younger patients had higher odds of presenting with *PIK3CA, SMAD3*, and *TSC2* somatic variations and decreased odds of presenting with *GNAS* variations compared with late-onset AC cases. These findings demonstrate that ACs diagnosed among young individuals harbor a distinct molecular phenotype compared with late-onset ACs and yield clinical actionability in future studies that should aim to elucidate distinct molecular phenotypes and mechanisms of early-onset AC and to develop and test personalized therapeutic modalities tailored to young patients diagnosed with AC.

## Notes

Supplement. **eTable 1. **Median Read Depth for Clinical-Grade Targeted Sequencing Data From Tumor Tissues and AC Case Counts by Sequencing Center **eTable 2. **Baseline Variation Probability and Differential Expression of Somatic Cancer Gene Variations by Age at Disease Onset (<50 vs ≥70 Years) Among Adults Diagnosed With Appendiceal Cancers **eTable 3. **Differential Expression of Nonsilent GNAS Variations Between Early-Onset (Age <50 Years) and Late-Onset (age ≥50 Years) Cases Diagnosed With Mucinous and Nonmucinous Adenocarcinomas of the Appendix
