# Error in Reporting of Age Variable in Database

**DOI:** 10.1001/jamanetworkopen.2021.6703

**Published:** 2021-03-26

In the Original Investigation titled “Spectrum of Somatic Cancer Gene Variations Among Adults With Appendiceal Cancer by Age,”^1^ published December 9, 2020, the age variable collected by the American Association for Cancer Research Project Genomics Evidence Neoplasia Information Exchange (GENIE) that was used in this study was reported incorrectly as the age at cancer diagnosis. The GENIE database includes the age at date of genomic sequencing, not the age at diagnosis. The article has been corrected to replace “disease onset” with “clinical sequencing.”^1^
