# Supplementary material for: Spectrum of Somatic Cancer Gene Variations Among Adults With Appendiceal Cancer by Age at Disease Onset
Source: JAMA Netw Open. 2020 Dec 9;3(12):e2028644. doi: 10.1001/jamanetworkopen.2020.28644 (PMC7726634; doi:10.1001/jamanetworkopen.2020.28644)
Supplement: Supplement. — eTable 1. Median Read Depth for Clinical-Grade Targeted Sequencing Data From Tumor Tissues and AC Case Counts by Sequencing Center eTable 2. Baseline Variation Probability and Differential Expression of Somatic Cancer Gene Variations by Age at Disease Onset (<50 vs ≥70 Years) Among Adults Diagnosed With Appendiceal Cancers eTable 3. Differential Expression of Nonsilent GNAS Variations Between Early-Onset (Age <50 Years) and Late-Onset (age ≥50 Years) Cases Diagnosed With Mucinous and Nonmucinous Adenocarcinomas of the Appendix [file jamanetwopen-e2028644-s001.pdf]

Supplemental Online Content

Holowatyj AN, Eng C, Wen W, Idrees K, Guo X. Spectrum of somatic cancer gene variations among adults with appendiceal cancer by age at disease onset. *JAMA Netw Open*. 2020;3(12):e2028644. doi:10.1001/jamanetworkopen.2020.28644

eTable 1. Median Read Depth for Clinical-Grade Targeted Sequencing Data From Tumor Tissues and AC Case Counts by Sequencing Center
eTable 2. Baseline Variation Probability and Differential Expression of Somatic Cancer Gene Variations by Age at Disease Onset (<50 vs ≥70 Years) Among Adults Diagnosed With Appendiceal Cancers
eTable 3. Differential Expression of Nonsilent GNAS Variations Between Early-Onset (Age <50 Years) and Late-Onset (age ≥50 Years) Cases Diagnosed With Mucinous and Nonmucinous Adenocarcinomas of the Appendix

This supplemental material has been provided by the authors to give readers additional information about their work.

© 2020 Holowatyj AN et al. *JAMA Network Open.*

eTable 1. Median Read Depth for Clinical-Grade Targeted Sequencing Data From Tumor Tissues and AC Case Counts by Sequencing Center

Sequencing Center	Median Sequencing Depth (X)	Total		Age of Appendiceal Cancer Onset			
				Early-Onset (<50 years)		Late-Onset (50+ years)	
		N	%	N	%	N	%
**Total**	500	385		109	28.3	276	71.7
Dana-Farber Cancer Institute	350	67	17.4	14	12.8	53	19.2
Duke Cancer Institute	1000	6	1.6	1	0.9	5	1.8
Johns Hopkins Sidney Kimmel Comprehensive Cancer Center	500	5	1.3	0	0.0	5	1.8
The University of Texas MD Anderson Cancer Center	250	30	7.8	8	7.3	22	8.0
Memorial Sloan Kettering Cancer Center	750	193	50.1	59	54.1	134	48.6
Netherlands Cancer Institute	4000	14	3.6	2	1.8	12	4.3
Providence Health & Services	500	1	0.3	0	0.0	1	0.4
Swedish Cancer Institute	200	10	2.6	2	1.8	8	2.9
University of Chicago Comprehensive Cancer Center	NA	2	0.5	0	0.0	2	0.7
Princess Margaret Cancer Centre	500	12	3.1	7	6.4	5	1.8
Vanderbilt-Ingram Cancer Center	1000	25	6.5	11	10.1	14	5.1
Wake Forest University Health Sciences	500	20	5.2	5	4.6	15	5.4

Abbreviations: NA, data not available.

© 2020 Holowatyj AN et al. *JAMA Network Open.*

eTable 2. Baseline Variation Probability and Differential Expression of Somatic Cancer Gene Variations by Age at Disease Onset (<50 vs ≥70 Years) Among Adults Diagnosed With Appendiceal Cancers

Odds ratios (OR) and 95% confidence intervals (CI) were calculated for genes from models adjusted for patient sex, race/ethnicity, histological subtype, sequencing center, and sample type. Reference outcome category was individuals age 70+ years at appendiceal cancer diagnosis. Genes ranked by baseline probability of mutation occurrence among individuals age <50 years at appendiceal cancer diagnosis. Bold text indicates *P*<0.05.

Gene Symbol	Baseline Mutation Probability by Age of Appendiceal Cancer Onset				OR	95% CI			*P*
	<50 years	50-59 years	60-69 years	70+ years					
KRAS	0.5229	0.4880	0.4947	0.5893	0.70	0.32	-	1.52	0.37
TP53	0.3303	0.2320	0.2553	0.2857	1.35	0.62	-	2.93	0.44
GNAS	**0.1927**	**0.2640**	**0.2766**	**0.3750**	**0.26**	**0.11**	**-**	**0.63**	**0.003**
PIK3CA	**0.1193**	**0.0240**	**0.0842**	**0.0179**	**11.69**	**1.37**	**-**	**99.82**	**0.02**
SMAD4	0.1193	0.1680	0.1170	0.1071	1.22	0.40	-	3.69	0.73
SOX9	0.0889	0.0417	0.1081	0.0784	1.28	0.34	-	4.81	0.72
KMT2D	0.0762	0.0446	0.0595	0.0192	7.49	0.73	-	76.48	0.09
APC	0.0734	0.0560	0.1579	0.0179	4.92	0.57	-	42.09	0.15
ATM	0.0734	0.0400	0.0426	0.0179	5.27	0.50	-	56.00	0.17
SMAD2	0.0667	0.0446	0.0357	0.0577	1.52	0.32	-	7.28	0.60
RNF43	0.0619	0.0309	0.0256	0.0392	1.17	0.19	-	7.01	0.86
SMAD3	0.0556	0.0104	0.0270	0.0	-				
NOTCH3	0.0515	0.0206	0.0128	0.0	-				
SETD2	0.0476	0.0089	0.0238	0.0	-				
MED12	0.0412	0.0000	0.0253	0.0196	1.29	0.11	-	14.66	0.84
ARID1A	0.0381	0.0714	0.0357	0.0577	0.60	0.12	-	3.08	0.54
TSC2	0.0381	0.0179	0.0119	0.0	-				
ERBB2	0.0367	0.0080	0.0105	0.0179	2.56	0.22	-	29.68	0.45
FBXW7	0.0367	0.0080	0.0532	0.0536	0.66	0.12	-	3.60	0.63
FAT1	0.0333	0.0417	0.0541	0.0392	0.79	0.11	-	5.60	0.81
TGFBR2	0.0330	0.0667	0.0606	0.0513	0.80	0.10	-	6.08	0.83
TCF7L2	0.0323	0.0000	0.0526	0.0	-				
PLCG2	0.0309	0.0206	0.0385	0.0	-				
ATRX	0.0286	0.0177	0.0119	0.0566	0.37	0.04	-	3.06	0.36
KDM6A	0.0286	0.0354	0.0238	0.0	-				

© 2020 Holowatyj AN et al. *JAMA Network Open.*

ASXL1	0.0280	0.0085	0.0349	0.0182	1.73	0.13	-	23.72	0.68
RB1	0.0275	0.0080	0.0213	0.0536	0.12	0.01	-	1.48	0.10
NOTCH1	0.0275	0.0400	0.0106	0.0357	0.67	0.07	-	6.35	0.73
EP300	0.0190	0.0179	0.0238	0.0577	0.38	0.04	-	3.72	0.41
CARD11	0.0190	0.0268	0.0119	0.0192	0.84	0.07	-	10.08	0.89
CDH1	0.0183	0.0320	0.0426	0.0714	0.21	0.02	-	1.74	0.15
CTNNB1	0.0183	0.0160	0.0000	0.0536	0.39	0.06	-	2.57	0.33
NRAS	0.0183	0.0320	0.0105	0.0179	1.44	0.12	-	17.29	0.77
BRAF	0.0183	0.0320	0.0421	0.0179	0.72	0.05	-	10.92	0.81
BCOR	0.0103	0.0102	0.0513	0.0385	0.24	0.01	-	4.21	0.33
ARID2	0.0095	0.0268	0.0476	0.0385	0.29	0.02	-	3.51	0.33
FLT1	0.0095	0.0268	0.0238	0.0192	1.12	0.05	-	23.42	0.94
ALK	0.0092	0.0160	0.0526	0.0179	0.31	0.01	-	7.39	0.47
AKT1	0.0092	0.0160	0.0316	0.0179	0.47	0.02	-	11.65	0.64

© 2020 Holowatyj AN et al. *JAMA Network Open.*

eTable 3. Differential Expression of Nonsilent GNAS Variations Between Early-Onset (Age <50 Years) and Late-Onset (age ≥50 Years) Cases Diagnosed With Mucinous and Nonmucinous Adenocarcinomas of the Appendix

Odds ratios (OR) and 95% confidence intervals (CI) were calculated for genes from models adjusted for patient sex, race/ethnicity, sequencing center, and sample type. Reference outcome category was individuals with late-onset appendiceal cancers.

Gene Symbol	AC Histological Subtype	OR	95% CI			*P*
GNAS	Mucinous Adenocarcinoma	0.35	0.15	-	0.79	0.01
	Non-Mucinous Adenocarcinoma	0.28	0.07	-	1.14	0.08

© 2020 Holowatyj AN et al. *JAMA Network Open.*
